# Endotracheal intubation using the C-MAC^® ^video laryngoscope or the Macintosh laryngoscope: A prospective, comparative study in the ICU

**DOI:** 10.1186/cc11384

**Published:** 2012-06-13

**Authors:** Ruediger R Noppens, Stephanie Geimer, Nicole Eisel, Matthias David, Tim Piepho

**Affiliations:** 1 Department of Anesthesiology, University Medical Center of the Johannes Gutenberg-University, Langenbeckstraße 1, 55131 Mainz, Germany

## Abstract

**Introduction:**

Endotracheal intubation in the ICU is a challenging procedure and is frequently associated with life-threatening complications. The aim of this study was to investigate the effect of the C-MAC^® ^video laryngoscope on laryngeal view and intubation success compared with direct laryngoscopy.

**Methods:**

In a single-center, prospective, comparative before-after study in an anesthetist-lead surgical ICU of a tertiary university hospital, predictors of potentially difficult tracheal intubation, number of intubation attempts, success rate and glottic view were evaluated during a 2-year study period (first year, Macintosh laryngoscopy (ML); second year, C-MAC^®^).

**Results:**

A total of 274 critically ill patients requiring endotracheal intubation were included; 113 intubations using ML and 117 intubations using the C-MAC^® ^were assessed. In patients with at least one predictor for difficult intubation, the C-MAC^® ^resulted in more successful intubations on first attempt compared with ML (34/43, 79% vs. 21/38, 55%; *P *= 0.03). The visualization of the glottis with ML using Cormack and Lehane (C&L) grading was more frequently rated as difficult (20%, C&L grade 3 and 4) compared with the C-MAC^® ^(7%, C&L grade 3 and 4) (*P *< 0.0001).

**Conclusion:**

Use of the C-MAC^® ^video laryngoscope improved laryngeal imaging and improved the intubating success rate on the first attempt in patients with predictors for difficult intubation in the ICU setting. Video laryngoscopy seems to be a useful tool in the ICU where potentially difficult endotracheal intubations regularly occur.

## Introduction

Airway management for critically ill patients in locations other than the operating room is challenging and frequently associated with life-threatening complications. For example, the incidence of difficult endotracheal intubations is higher in the ICU than in the operating room. The number of difficult intubations ranges from 10 to 22% in critically ill patients [1-3]. Visualizing the glottis is often difficult in the ICU due to the constraints of space, the position of the patient and the accompanying comorbidities [4]. Additionally, multiple attempts of endotracheal intubation are often necessary to secure the patient's airway in the ICU setting and are known to increase the risk of life-threatening complications, such as severe hypoxia, esophageal intubation, aspiration and cardiac arrest [2,5,6]. This knowledge suggests that optimization of visualization of the glottis might reduce complications.

Video laryngoscopes seem promising for airway management [7]. Video laryngoscopes contain a small camera and a light source at the distal third of the blade. The video picture is transferred to a monitor. The C-MAC^® ^video laryngoscope (Karl Storz GmbH & Co. KG, Tuttlingen Germany) evaluated in this study uses Macintosh-shaped blades. Two approaches to visualize the glottis with the use of a Macintosh video laryngoscope blade are available: first, the direct view of the glottis; and second, an indirect view by means of a miniature camera on the screen of the monitoring unit. Several studies have shown the successful use of the C-MAC^® ^in the operating room and in prehospital emergency medicine [8,9]. The use of Macintosh blades with the C-MAC^® ^improved the glottic view in patients who were difficult to intubate using direct laryngoscopy in the operating room [10]. These data cannot be directly translated to the situation on the ICU, because performing endotracheal intubation is more challenging in this environment.

The aim of this study was to evaluate the glottic view, number of intubation attempts and success rate of endotracheal intubation in an anesthetist-lead surgical ICU using Macintosh laryngoscopy (ML) or the C-MAC^® ^video laryngoscope. Additionally, we evaluated whether the level of physician experience might influence visualization of the glottis or intubation success. We hypothesized that the use of a video laryngoscope would improve the glottic view and reduce the number of intubation attempts.

## Materials and methods

The ethical committee of the medical association of the State of Rhineland-Palatinate approved the study and the committee waived the need for specific written informed consent. The study was performed in a 21-bed anesthetist-lead adult surgical ICU of a tertiary-care university teaching hospital.

This prospective, comparative, before-after study evaluated the endotracheal intubations of critically ill patients over a 2-year period in the ICU. Participating physicians completed a standardized evaluation form immediately after performing an endotracheal intubation.

Predictors of potentially difficult tracheal intubation were recorded for each patient on the standardized evaluation form: short neck with large circumference, obesity, limited mouth opening (< 3 cm), limited neck movement, presence of a large tongue and a short thyromental distance.

The laryngoscopic view was evaluated using the Cormack & Lehane (C&L) classification scale [11] and the Percentage of Glottic Opening scale (POGO) [12]. The number of intubation attempts, intubation success, the indication for the intubation and possible complications were documented. The lowest oxygen saturation during intubation was measured using pulse oximetry. Age, gender, physical status, the Simplified Acute Physiology Score and the Sequential Organ Failure Assessment II score were collected for all patients in this study at admission into the ICU. The clinical experience of the laryngoscopist was also noted. Junior physician was defined with up to 3 years clinical experience, senior physician with more than 3 years of training, and specialists were board-certified anesthesiologists.

Over a 12-month evaluation period (January 2009 to January 2010), the standard procedures for tracheal intubation remained unchanged. Restrictions concerning the selection of airway management tools for endotracheal intubation did not exist (baseline). Direct laryngoscopy (ML) was performed using a size 3 or size 4 regular Macintosh blade. Alternative airway devices (for example, intubation endoscope, laryngeal mask airway, and cricothyrotomy set) were always available in an airway cart at the bedside. After three failed attempts at endotracheal intubation, alternative devices (for example, endoscopic intubation) were used according to the in-house difficult airway algorithm.

The presence of at least two healthcare professionals, with at least one senior physician, was standard for all intubations. If possible, pre-oxygenation for 3 minutes at a high constant flow or non-invasive positive-pressure ventilation was administered to the patients. The medication for the induction of anesthesia was sufentanil (0.3 to 1.0 μg/kg) in every patient, with either propofol (1.5 to 2.0 mg/kg), ketamine (1.5 to 3 mg/kg) or etomidate (0.2 to 0.3 mg/kg). Rocuronium (0.4 to 0.9 mg/kg) was always used for neuromuscular blockade. In cardiac arrest patients, intubations were performed without medication. A malleable stylet in a hockey-stick shape was always used for tube placement. If visualization of the glottis or the placement of the endotracheal tube was difficult, the manipulation of the larynx was performed according to the instructions of the laryngoscopist. The successful placement of the endotracheal tube was confirmed using capnography.

**Figure 1:**
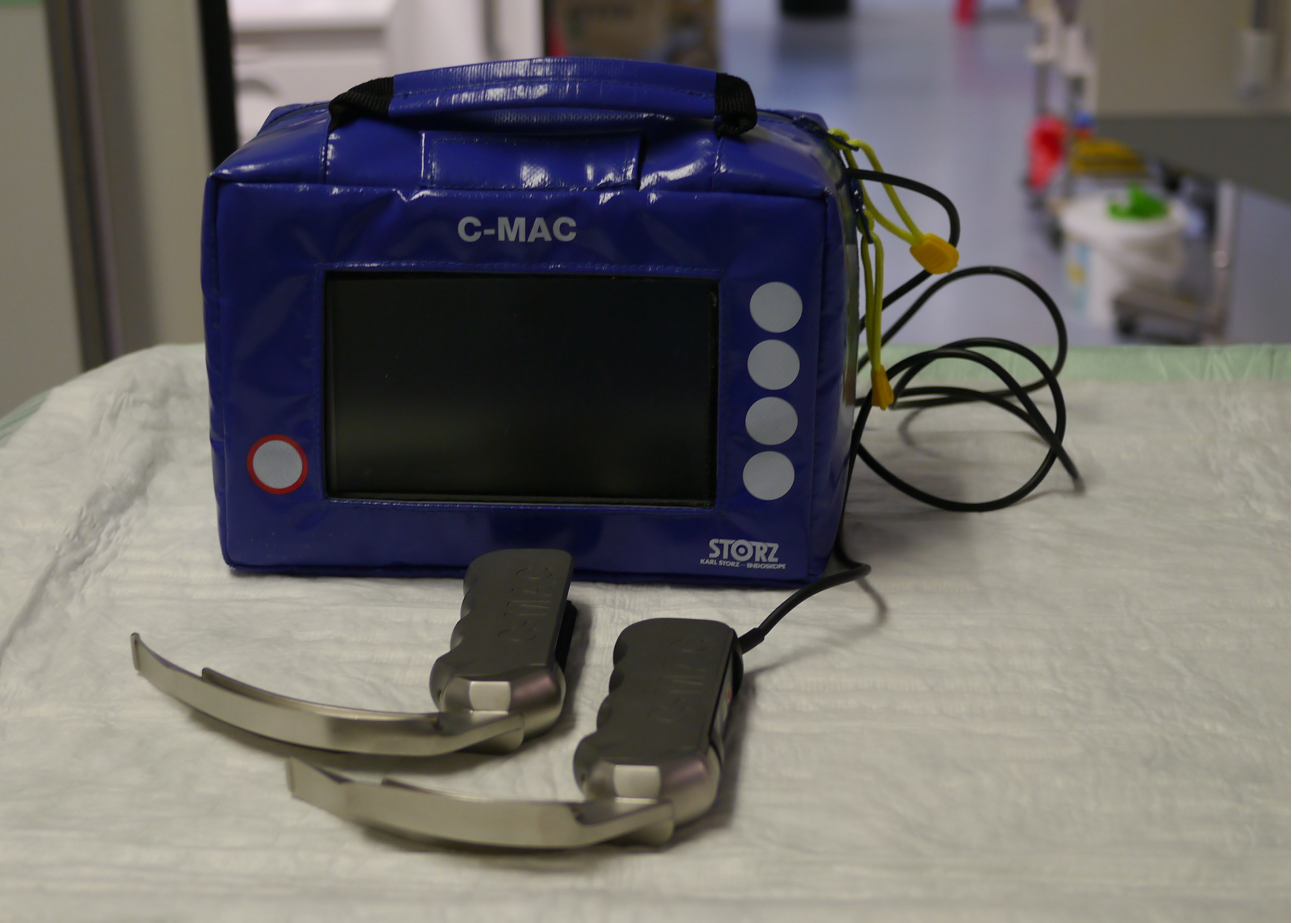
**The C-MAC^® ^video laryngoscope with size 3 and size 4 blades**.

We then evaluated endotracheal intubations over a second 12-month period (February 2010 to February 2011) after implementing two C-MAC^® ^video laryngoscopes (Karl Storz GmbH & Co. KG) in the ICU (intervention phase). Video laryngoscopy was performed using the Karl Storz Macintosh shaped blades for C-MAC^® ^size 3 or size 4 (Figure 1). ICU physicians were given didactic instruction on the proper use of the C-MAC^® ^along with training on manikins. The ICU staff were advised to perform endotracheal intubations using the C-MAC^® ^instead of ML when appropriate. The procedures for intubation, the medication for anesthesia and the in-house difficult airway algorithm were identical to the first evaluation period. Documentation was identical to the previous study period of 2009 to 2010.

Patients who presented with predictors of potentially difficult tracheal intubations were identified and analyzed in a subgroup for first-attempt intubation success and visualization of the glottis.

After use, the C-MAC^® ^blades and cable with the electronic module were manually cleaned and immersed in a cleaning solution (Teralin^®^; Schülke & Mayr, Norderstedt, Germany). Automated processing using an endoscope cleaning and disinfection unit was then used for disinfection (BHT 2000^®^; BHT Hygiene Technik, Gersthofen, Germany). The monitor unit was cleaned manually using disinfection cloths (Mikrobac^®^; Bode, Hamburg, Germany).

Data were analyzed using GraphPad Prism (version 5a for MAC; GraphPad Software, San Diego, CA, USA). Data are expressed as the mean ± standard deviation and the median (interquartile range) for non-Gaussian variables. The comparison of the two proportions was performed with the use of the chi-square test or Fischer's exact test when appropriate. The comparison of means was performed using Student's *t *test, and comparison of the medians was performed with the Mann-Whitney test. One-way analysis of variance with Dunn's *post-hoc *test was used for multiple comparisons. The differences were considered statistically significant if *P *< 0.05.

## Results

**Figure 2:**
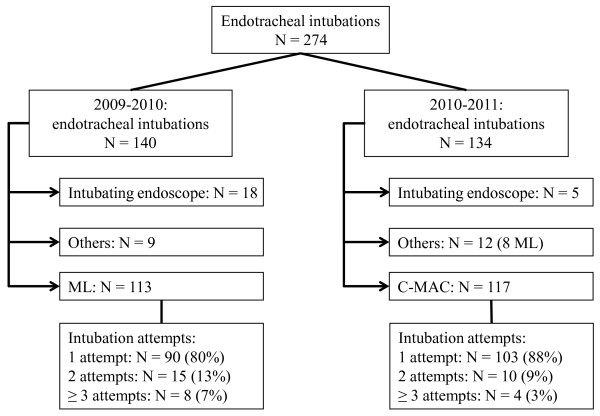
**Flow chart of the prospective study**. ML, Macintosh laryngoscopy.

A total of 274 patients were evaluated during the 2-year study period (Figure 2). There were no differences in their demographic variables (Table 1). Physical status was similar between groups (Simplified Acute Physiology Score); however, patients in the ML group presented with a higher Sequential Organ Failure Assessment II score (*P *< 0.05; Table 1). The indications for intubation did not differ between the ML and the C-MAC^® ^groups (Table 1).

**Table 1:** Demographic data, indication for endotracheal intubation, and level of laryngoscopist training during baseline and intervention

	Macintosh laryngoscopy (*n *= 140)	C-MAC^® ^video laryngoscope (*n *= 134)	*P *value
Age (years)	62.9 ± 15.6	63.8 ± 16.7	0.66
Gender (male/female)	87/53	86/48	0.73
Body mass index (kg/m^2^)	26.9 ± 7.6	26.7 ± 6.4	0.85
Sequential Organ Failure Assessment II score	8.7 ± 4.7	9.2 ± 4.5	0.40
Simplified Acute Physiology Score	38.2 ± 14.8	33.9 ± 13.6	0.03
Indication for endotracheal intubation			
Respiratory insufficiency	83	86	0.90
Reduced consciousness	8	9	1.0
Pre-interventional	9	7	0.61
Cardiopulmonary resuscitation	3	2	0.68
Others	13	2	0.003
Endotracheal tube change	11	22	0.06
Indication not documented	13	6	0.20
Experience of the laryngoscopist			
< 3 years	37	52	0.02
> 4 years	20	33	0.03
Specialist	83	49	0.0002

Data presented as mean ± standard deviation or *n*.

With the availability of the video laryngoscope, more physicians-in-training performed endotracheal intubations. In contrast, mainly specialists performed endotracheal intubations during the baseline phase (*P *< 0.001; Table 1).

During the baseline phase of the study, a total of 113 intubations were performed using ML. A total of 117 intubations were performed using the C-MAC^® ^during the intervention phase of the study. After the introduction of a video laryngoscope, the use of intubating endoscopy was used less frequently used for intubation compared with the baseline phase (5/134, 4% vs. 18/140, 13%; *P *< 0.05) (Figure 2).

After introduction of the C-MAC^®^, eight patients were intubated using ML. Five patients were intubated in one attempt, and three patients were intubated after two attempts. There were no differences in the number of intubation attempts, successful intubations and views of the glottis between junior, senior and specialist physicians (Table 2).

**Table 2:** Intubation attempts and visualization of the glottis using the Cormack & Lehane grade

	Attempts (*n*)	Cormack & Lehane grade
Macintosh laryngoscopy		
Junior	1 (1 to 1); 1 to 3	2 (1 to 2); 1 to 3
Senior	1 (1 to 1); 1 to 4	1 (1 to 2); 1 to 2
Specialist	1 (1 to 2); 1 to 4	1 (1 to 2); 1 to 4
*P *value	0.36	0.22
C-MAC^® ^video laryngoscope		
Junior	1 (1 to 1); 1 to 1	1 (1 to 2); 1 to 4
Senior	1 (1 to 1); 1 to 5	1 (1 to 1); 1 to 4
Specialist	1 (1 to 1); 1 to 3	1 (1 to 2); 1 to 4
*P *value	0.91	0.39
*P *value (ML vs. C-MAC^®^)		
Junior	0.54	0.14
Senior	0.60	0.98
Specialist	0.10	0.17

Data presented as median (interquartile range); range. Junior, < 1 to 3 years of clinical experience; senior, > 3 years of clinical experience; specialist, board-certified anesthesiologist. ML, Macintosh laryngoscopy.

No differences in predictors for potential difficult intubation were observed (Table 3). In 15% of ML and 18% of C-MAC^® ^intubations, at least one predictor existed. The most often described predictor was a short neck with large circumference (Table 3).

**Table 3:** Predictors for difficult intubation, number of complications and lowest documented oxygen saturation during endotracheal intubation

	Macintosh laryngoscopy (*n *= 113)	C-MAC^® ^video laryngoscope (*n *= 117)	*P *value
Predictors for difficult intubation			
Short, big neck	23 (20%)	24 (21%)	0.97
Obesity	16 (14%)	17 (15%)	0.93
Limited mouth opening	5 (4%)	6 (5%)	0.80
Limited neck movement	13 (12%)	14 (12%)	0.91
Large tongue	2 (2%)	9 (8%)	0.04
Short thyromental distance	6 (5%)	9 (7%)	0.46
1 predictor	17 (15%)	21 (18%)	0.55
2 predictors	13 (12%)	10 (9%)	0.45
> 2 predictors	7 (6%)	12 (10%)	0.26
Complications			
Minor tissue injury	3	1	
Bleeding	3	2	
Regurgitation/aspiration	1	0	
Glottic swelling	1	4	
Endobronchial intubation	0	3	
Esophageal intubation	2	0	
Other	7	4	
Total events	17 (15%)	14 (12%)	0.70
Lowest SpO_2 _during intubation			
< 80%	14 (12%)	15 (13%)	0.92
80 to 90%	27 (24%)	28 (24%)	0.99
> 90%	72 (64%)	74 (63%)	0.94

Data presented as *n *(%). SpO_2_, oxygen saturation.

Complications during endotracheal intubations occurred in 17 (12%) cases in the baseline phase of the study and in 14 (10%) patients after the C-MAC^® ^was introduced in the intervention phase of the study (*P *= 0.7; Table 3). The types of complications did not differ between the groups. The oxygenation saturation as measured by pulse oximetry did not differ between groups (Table 3).

The number of attempts needed for securing the airway of patients was not different between the ML and the C-MAC^® ^groups (*P *= 0.21; Figure 2). The rate for difficult intubation (using the definition of at least two failed intubation attempts) was 7% and 3% in the ML and C-MAC^® ^groups, respectively (Figure 2). The rate of success for the first intubation attempt did not differ when the C-MAC^® ^was used compared with ML (103/117, 88% vs. 89/113, 79%; *P *= 0.08). If at least one predictor for potential difficult intubation was present, the success rate for endotracheal intubation at the first attempt was higher (34, 79%) in the C-MAC^® ^group compared with the ML group (22, 56%; *P *= 0.03) (Table 4).

**Table 4:** Patients presenting with at least one potential predictor for difficult intubation

	Macintosh laryngoscopy (*n *= 39)	C-MAC^® ^video laryngoscope (*n *= 43)	*P *value
Success rate at first attempt	22 (56%)	34 (79%)	0.03
Cormack & Lehane grade	2 (1 to 3); 1 to 4	1 (1 to 2); 1 to 4	0.003
Grade I	10	26	
Grade II	14	9	
Grade III	7	4	
Grade IV	8	4	
Percentage of glottic opening	42 ± 36	76 ± 32	< 0.001

Data presented as *n *(%), median (interquartile range); range or mean ± standard deviation.

**Figure 3:**
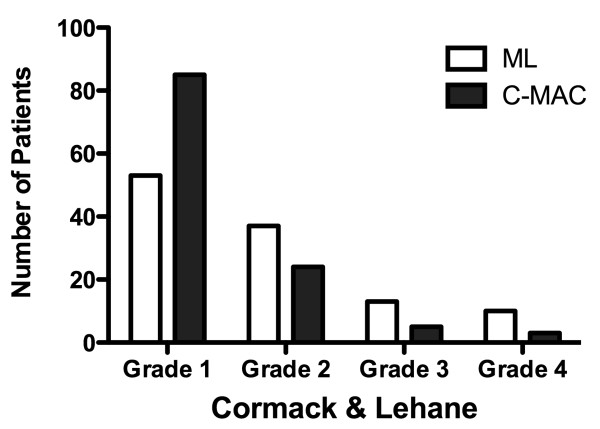
**Visualization of the glottis using Cormack & Lehane classification**. ML, Macintosh laryngoscopy. *P *< 0.0001.

Using the C&L grading scale, the visualization of the glottis with ML was more frequently rated as difficult (Figure 2). The glottis could not be visualized in 23 patients (20%, C&L grade 3 or 4), was only partially visualized in 37 patients (33%, C&L grade 2) and was fully visualized in 53 patients (47%, C&L grade 1) (Figure 3). In contrast, visualization of the glottis was improved with the C-MAC^® ^(*P *< 0.0001): C&L grade 3 or 4 was rated in eight patients (7%), C&L grade 2 was rated in 24 patients (21%) and C&L grade 1 was rated in 85 patients (73%). The use of the C-MAC^® ^resulted in an improved view of the glottis as measured by the POGO scale compared with ML (60 ± 36% vs. 82 ± 25%; *P *< 0.001). The glottic view was improved using the C-MAC^® ^compared with ML when predictors for difficult intubation were present (C&L, *P *= 0.027; POGO, *P *< 0.001) (Table 4).

## Discussion

In this prospective study of 247 consecutive patients over a 2-year period, endotracheal intubation was associated with a high rate of difficult laryngeal visualization and a high number of repeated intubation attempts. The use of the C-MAC^® ^video laryngoscope improved visualization of the glottis during airway management in the ICU. Patients with a potential difficult airway had a higher success rate for intubation at the first attempt when the video laryngoscope was used.

The major advantage of video laryngoscopes is that the glottis can be visualized indirectly via a screen without a direct line of view (look around the corner). One potential problem is that the tip of the endotracheal tube has to pass a sharp angle to enter the larynx, which increases the risk of contact with the anterior tracheal wall. As a result, the tube cannot be easily advanced into the trachea. This phenomenon has been described with the use of several video laryngoscopes, such as the McGrath Series 5^® ^(Aircraft Medical Ltd, Edinburgh, UK) and the GlideScope^® ^(Verathon Inc., Bothell, WA, USA). The use of a video laryngoscope with a Macintosh shaped video blade reduced the problem of tube advancement despite a good glottic view compared with the video laryngoscopes that use a more curved blade. In a comparison of the use of ML versus the use of the C-MAC^® ^in groups of patients who had a difficult laryngoscopy during a scheduled surgical procedure, the use of the C-MAC^® ^improved the glottic view in 94% (49/52) of patients [10]. In the operating room, use of the C-MAC^® ^in patients with a predicted difficult airway improved optical access to the glottis compared with direct laryngoscopy using a Macintosh laryngoscope and resulted in more successful intubations at the first attempt [13].

Little is known about the effect of using a video laryngoscope in the challenging ICU environment. In a small study examining the effect of the GlideScope^® ^video laryngoscope, no effect was observed on the number of intubation attempts and the occurrence of complications [14]. In the emergency department, no difference in intubation success between the GlideScope^® ^and direct laryngoscopy was described [15]. In the presence of a difficult airway situation, however, use of the video laryngoscope resulted in a higher success rate compared with direct laryngoscopy [15]. Despite numerous promising studies, not all instruments using indirect laryngoscopy were found to be efficient in settings outside the operating room. In a prehospital randomized study, the use of the Airtraq^® ^laryngoscope (Podol Lt., Vizcaya, Spain) was associated was a high incidence of failed intubations compared with direct laryngoscopy using a Macintosh laryngoscope [16]. In our study, the use of the C-MAC^® ^only resulted in a more successful intubation at the first attempt in predicted difficult airways, compared with the use of ML. The overall success rate of the C-MAC^® ^was similar to ML when all endotracheal intubations were analyzed.

After the introduction of the C-MAC^®^, specialist anesthesiologists less frequently performed endotracheal intubation personally. However, this had no effect on the glottic view, the number of intubation attempts and the number of complications.

Difficult intubation is a rather common event in locations other than the operating room. Recent studies have stated that the occurrence of difficult intubations in critically ill patients ranges from 10 to 22% [1-3]. In our study, the rate of difficult laryngoscopy (C&L grade 3 and 4) using ML was 20%. This is remarkable since the large majority of physicians participating in this study were anesthesiologists. A French study reported a similar incidence of difficult laryngoscopy [3]. The incidence of difficult laryngoscopies reported for the operating room (5%) is significantly less frequent than in other settings [17]. In a recent report from the UK, more than 60% of the events associated with airway management in the ICU led to death or brain damage [18]. The corresponding incidence in the operating room was 14% [19]. The possible reasons for the high rate of severe complications outside the operating room are probably patient-related factors and include multiple organ failure, advanced age, the use of vasopressors and low fluid responsiveness [1].

### Limitations

The major limitation of this work is that a nonrandomized study design was used. This might have resulted in a higher Sequential Organ Failure Assessment II score in the ML group and heterogeneity of the training level of the laryngoscopists. We decided on a before-after design because we did not have the resources to guarantee patient randomization throughout the 2-year study period in our multiple-floor ICU. This study was planned in 2008, when few data were available about performance of video laryngoscopes in patients. This manuscript gives a very good background for future study designs, sample calculations, and so forth. More than 400 patients probably need to be included in a future study to show the effects on intubation success for the whole ICU population. Another possible limitation of this study was the subjective nature of the assessment of the laryngeal view completed by the participating physicians using the C&L grading system and the POGO score. The reproducibility of laryngeal view grading in anesthesiologists familiar with this classification is limited [20]. Evaluated data are self-reported, so a recall or reporting bias might be present.

Most of the studies that analyze advanced airway management tools are conducted by experienced operators, which could lead to a bias in favor of the choice of the instrument analyzed. Our study was conducted in a real-life setting, and the participating physicians represented a typical range of anesthesiologists.

## Conclusions

With the presence of at least one predictor for difficult intubation, a high incidence of an insufficient glottic view and repeated intubation attempts during airway management was documented in the ICU. The introduction of the C-MAC^® ^video laryngoscope improved the laryngeal view during intubation. In patients with the presence of a predicted difficult airway, use of the C-MAC^® ^resulted in more successful intubations on the first attempt. C-MAC^® ^video laryngoscopy using a Macintosh shaped blade seems to be a useful technique as the initial approach for endotracheal intubation in the ICU. These data justify larger randomized studies to evaluate the impact of video laryngoscopy on patient outcome.

## Key messages

• In this study, 15 to 18% of patients presented with at least one predictor for difficult intubation.

• Difficult visualization of the vocal cords using direct ML is common during endotracheal intubation in the ICU.

• Use of the C-MAC^® ^video laryngoscope improved visualization during endotracheal intubation.

• Using the C-MAC^® ^video laryngoscope for intubation reduced the number of intubating attempts if at least one predictor for difficult intubation was present.

## Abbreviations

C&L: Cormack & Lehane grading; ML: Macintosh laryngoscopy; POGO: percentage of glottis opening.

## Competing interests

Neither the Department of Anesthesiology of the University Medical Centre of the Johannes Gutenberg University-Mainz, Germany or any of its employees received any compensation for this work. Airway Hands-on Workshops and studies of the Department of Anesthesiology have been supported by Aircraft Medical Ltd (Edinburgh, UK), Ambu GmbH (Bad Nauheim, Germany), Karl Storz GmbH & Co. KG (Tuttlingen, Germany), The Surgical Company GmbH (Kleve, Germany) and Verathon Medical (Rennerod, Germany). No other funding or competing interests declared. The authors alone are responsible for the content and writing of the paper.

## Authors' contributions

RRN participated in the study design, data analysis, interpretation of the data and writing of the manuscript. SG and NE participated in the study design, and performed data acquisition and data analysis. MD participated in the study design and revised the manuscript. TP participated in the study design, data analysis and interpretation of the data, and revised the manuscript. All authors read and approved the manuscript for publication.
